# The Effect of Thunder-Fire Moxibustion on Lumbar Disc Herniation: Study Protocol for a Randomized Controlled Trial

**DOI:** 10.3389/fpubh.2022.930830

**Published:** 2022-07-06

**Authors:** Jiale Zhang, Xu Zhai, Xue Wang, Liuqing Wang, Hongxuan Tong, Tiancai Xian, Lexuan Shao

**Affiliations:** ^1^Institute of Basic Theory for Chinese Medicine, China Academy of Chinese Medical Sciences, Beijing, China; ^2^Graduate School, China Academy of Chinese Medical Sciences, Beijing, China; ^3^Department of Acupuncture and Massage, Nanling Hospital of Traditional Chinese Medicine, Wuhu, China; ^4^Institute for the History and Literature of Traditional Chinese Medicine, China Academy of Chinese Medical Sciences, Beijing, China; ^5^Genius Tianzhiyuan Group Co., Ltd., Ningbo, China

**Keywords:** complementary medicine, moxibustion, lumbar disc herniation, protocol, randomized controlled trial

## Abstract

**Background:**

Lumbar disc herniation (LDH) is a common disease seen in orthopedics; it is caused by nucleus pulposus herniation. Its clinical manifestations are low back pain, radiating pain of the lower limbs, and cauda equina symptoms that seriously affect patients' quality of life. At present, oral analgesics are commonly used in the treatment of LDH; but they can produce gastrointestinal reactions and other side effects. Thunder-fire moxibustion is a method that is widely used in China to treat pain syndromes. This study aimed to design a randomized controlled trial to explore the effectiveness and safety of thunder-fire moxibustion in the treatment of lumbar disc herniation.

**Methods:**

Ninety patients will be enrolled and randomly divided into one of two groups: the thunder-fire moxibustion group and the acetaminophen group. The thunder-fire moxibustion group will be treated with moxa sticks at BL25, GV3, BL23, and GV4; and after 15 min of local whirling moxibustion, the contralateral acupoints will be treated with moxibustion for 15 min. The study period will include two 10-day courses of treatment, for a total study duration of 20 days. The acetaminophen group participants will take one acetaminophen sustained-release tablet twice a day for the duration of the study period. In contrast, the thunder-fire moxibustion group participants will be treated with thunder-fire moxibustion every other day for 30 min. The primary outcome will be the Japanese Orthopedic Association (JOA) score. Visual analog scale (VAS) and Oswestry Disability Index (ODI) will be used as the secondary outcome measures. Adverse events (AEs) will also be recorded. Assessments will be conducted at baseline, the end of the first and second courses of treatment.

**Discussion:**

This study will determine whether thunder-fire moxibustion is more effective and safer than acetaminophen in the treatment of patients with LDH.

**Trial Registration:**

Chinese Clinical Trial Registry (http://www.chictr.org.cn), ChiCTR2000036079.

## Introduction

Lumbar disc herniation (LDH) is one of the most common causes of low back pain (1). It is a syndrome caused by the degeneration of the lumbar intervertebral disc, the rupture of the annulus fibrosus, the protrusion of nucleus pulposus tissue, and the stimulation of the lumbosacral nerve root and cauda equina nerve (2). From 1999 to 2013, 188 countries around the world investigated the prevalence of 301 kinds of acute and chronic diseases. They found that low back pain and depressive disorder were among the top 10 causes of disability in every country (3). At present, the treatment strategies for LDH include surgical and non-surgical treatment, and non-surgical treatment includes acupuncture and moxibustion, traction, drugs, functional exercise, and physical therapy (4, 5).

In treating LDH, oral non-steroidal anti-inflammatory drugs (NSAIDs) can be used as the first-line drugs for acute and chronic pain. Acetaminophen and NSAIDs have been recommended as the first-line medication options for most patients with low back pain (grade 1A evidence) (6). The efficacy of acetaminophen in the treatment of LDH has also been reported, (7) but it only relieves pain and fails to relieve the other symptoms of LDH.

Acupuncture, massage, and other complementary therapies have been widely used in the treatment of lumbar disc herniation (8–10). Among them, thunder-fire moxibustion, an important alternative therapy, plays a key role in promoting peripheral blood circulation and accelerating the absorption of inflammatory exudates (11, 12). The penetration of thunder-fire moxibustion is stronger than that of general moxibustion (13), especially in duration, which is longer than ordinary moxa sticks, with wide coverage and strong potency. Thunder-fire moxibustion sticks are composed of moxa and other natural Chinese herbal medicine, which can activate blood circulation, relieve blood stasis and reduce pain (14). In previous clinical trials, the experimental groups receiving thunder-fire moxibustion for LDH were mostly receiving thunder-fire moxibustion in combination with other treatment methods (15–17). To date, no relevant clinical trials have evaluated the specific effects of thunder-fire moxibustion alone in treating LDH. Thus, this study will be to evaluate efficacy and safety of thunder-fire moxibustion, as opposed to acetaminophen, in the treatment of patients with LDH.

## Methods

### Study Design

A single-center randomized controlled trial (RCT) will be designed to compare the efficacy and safety of thunder-fire moxibustion with that of acetaminophen in treating LDH. Participants in the acetaminophen group will receive twenty treatments over the 20-day study period. Each treatment will consist of two acetaminophen sustained-release tablets and will be administered to participants. Twice a day, one tablet at a time. Meanwhile, the participants in the thunder-fire moxibustion group will be treated with thunder-fire moxibustion once every other day for 30 min per treatment session.

**Figure 1:**
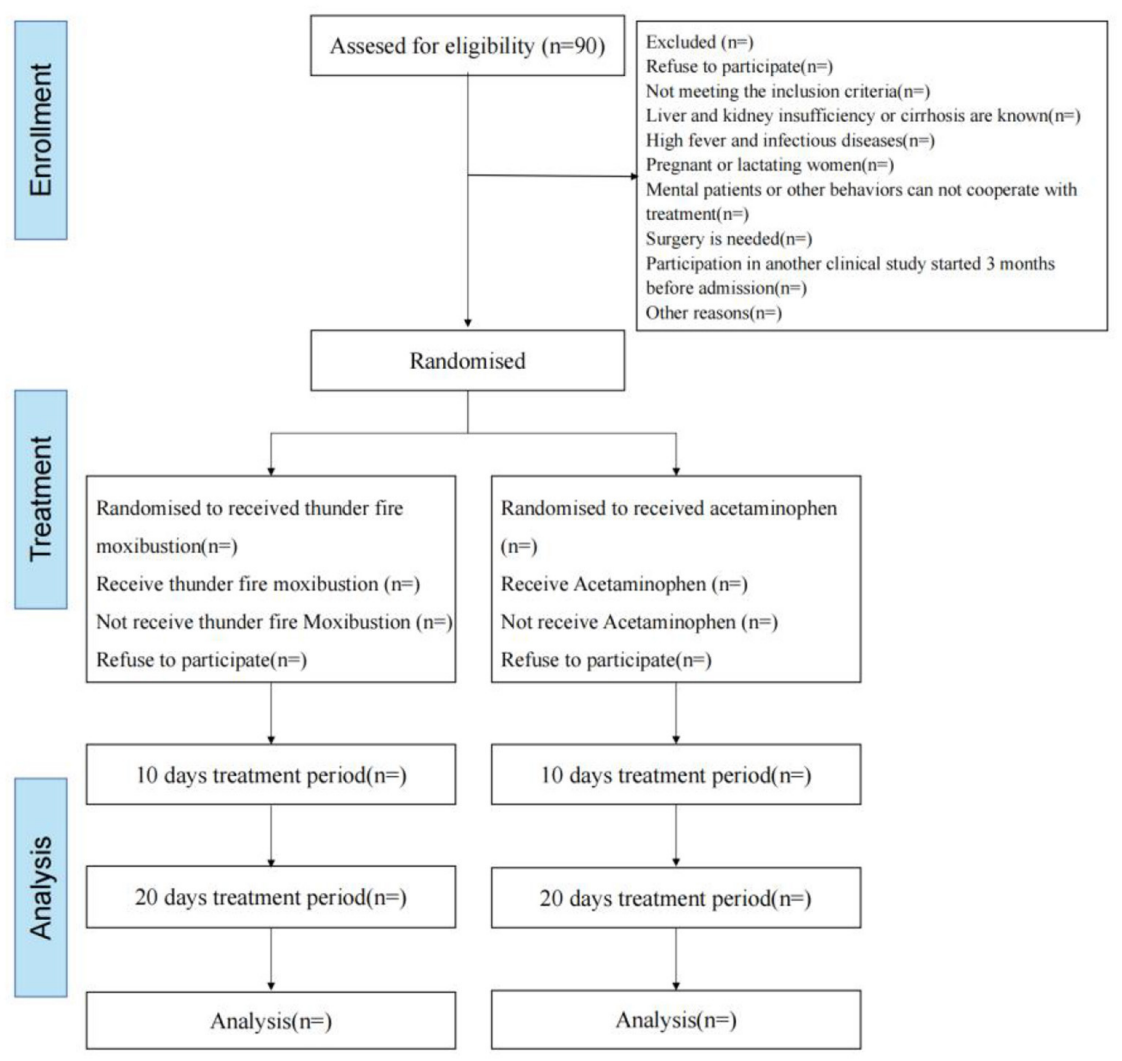
Flow chart of the trial. A total of 90 participants will be randomized to the two groups. The study period will consist of the baseline, end of the 10-day intervention, and 20-day intervention.

In this study, 90 LDH patients will be randomly divided into either the thunder-fire moxibustion group or the acetaminophen group at a ratio of 1:1 (Figure 1). At the same time, the male-female ratio of the two groups is 1:1. Our study will follow the general clinical trial rules as defined by the Helsinki Declaration. The SPIRIT checklist is given in Supplementary Material 1.

### Inclusion Criteria

(1) The age of the participants ranges from 25 to 80 years old. (2) The participant meets the diagnostic criteria of LDH as defined by the criteria in “Diagnosis and therapeutic effect of diseases and syndromes in traditional Chinese medicine” (18) and “An evidence-based clinical guideline for the diagnosis and treatment of lumbar disc herniation with radiculopathy” (19). (3) The clinical manifestations and imaging findings of the participants should be consistent with LDH. (4) Participants must provide signed informed consent.

### Exclusion Criteria

(1) Participants with severe heart, brain, liver, and haematopoietic system diseases or other serious diseases that affect survival.
(2) Participants with hepatic and renal insufficiency or cirrhosis.
(3) Participants with high fever and infectious diseases.
(4) Pregnant or lactating women.
(5) Participants with mental illness or those who cannot cooperate with the treatment.
(6) Participants with allergies or suspected allergies to the treatments in this trial.
(7) Participants with severe LDH who need surgical treatment.
(8) Participants in another clinical study in the past 3 months, and there are potential risks affecting this trial.

### Recruitment

We will recruit participants by advertising on bulletin boards located at the Department of Acupuncture and Massage, Nanling Hospital of Traditional Chinese Medicine. Staff working in this department will recruit participants based on the inclusion and exclusion criteria. Participant information will be kept by the data monitoring committee (DMC) and will not be disclosed to any individuals or organizations unrelated to this study.

### Random Assignment

A simple randomization method will be used. SPSS software (version 24.0) will be used to create the randomization sequence. Participants with LDH will be randomly assigned to either the thunder-fire moxibustion group or the acetaminophen group in a 1:1 ratio. The random assignment list generated will be placed in opaque sealed envelopes and distributed to the participants by a researcher (LX Shao).

### Blinding

This is an open-label trial; neither the participants nor the acupuncturists will be blinded to the treatment. All assessors, data recorders, and statisticians will operate independently; the randomization staff and acupuncturists will have access to the patient allocation information, while the assessor and statistician will remain blind to this information throughout the study. Each participant will be treated separately to prevent any exchange of study information. In the case that withdrawal occurs, the study research assistant will provide the relevant information to the participant, which should include the participant's treatment assignment and outcome data.

### Interventions

**Figure 2:**
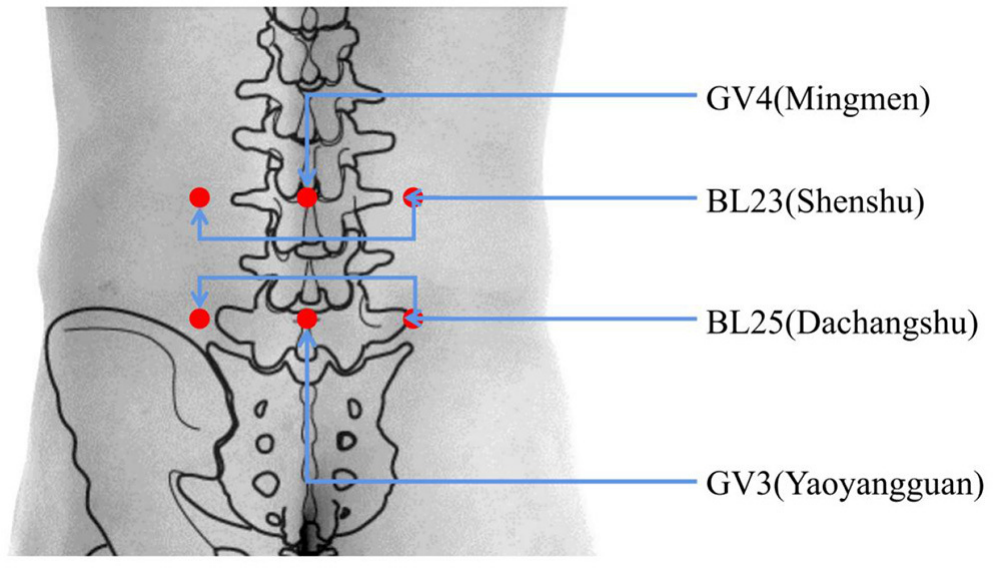
Location of acupoints. BL25 (Dachangshu); under the spinous process of the fourth lumbar spine, with a side opening of 5 cm. GV3 (Yaoyangguan); on the posterior midline, under the spinous process of the fourth lumbar spine. BL23 (Shenshu); under the spinous process of the second lumbar spine, with a side opening of 5 cm. GV4 (Mingmen); on the posterior midline, under the spinous process of the second lumbar spine.

The operators (acupuncturists) in this study all have more than 2 years of work experience and Chinese TCM doctor certificate, and will receive specific before the study period begins. The moxa sticks are produced by Nanyang Xiancao Industry Co., Ltd. Each moxa stick is 40 mm in diameter, 109 g in weight, and 15 cm in length. The treatment site is at the lower waist, where the acupoints BL25 (Dachangshu), GV3 (Yaoyangguan), BL23 (Shenshu), and GV4 (Mingmen) will be treated (Figure 2). In the quiet treatment room, the participant should be in a prone position. One moxa stick will be held by the operator who will ignite the top of the stick. Then, the moxa stick will be placed on the treatment site, and the fire head will be ~3 cm away from the skin. After 15 min of local whirling moxibustion, the contralateral acupoints will be treated with moxibustion for 15 min as well. During each treatment, the participant will feel a warm sensation but no burning pain. The frequency of the treatments will be every other day, five times in 10 days, for a total of 20 days, or 10 treatments. Participants in the acetaminophen group will receive two sustained-release acetaminophen tablets (0.65 g per tablet) (Paracetamol Sustained-release Tablets, Shanghai Johnson & Johnson Pharmaceutical Co. Ltd.). Participants take one tablet of acetaminophen every time for two times per day (0.65 g bid). After the trial, all participants will be offered six free acupuncture treatments or health counseling as compensation.

### Outcome Measurements

**Figure 3:**
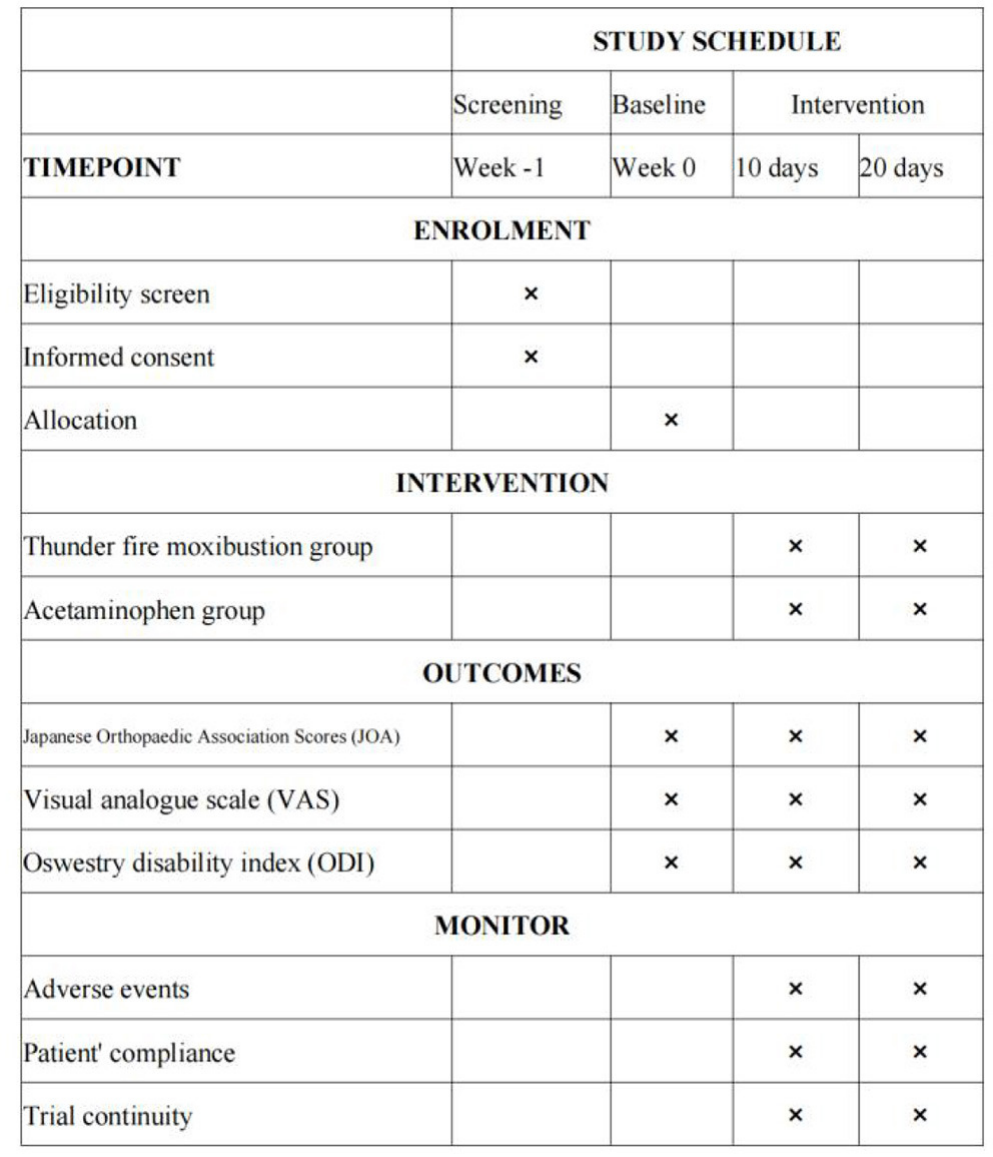
Study schedule. VAS, visual analog scale; ODI, oswestry disability index.

All outcome measurements will be recorded at baseline (before treatment), on day 10, and on day 20 (the conclusion of treatment) (Figure 3).

### Primary Outcome Measures

#### Japanese Orthopedic Association (JOA) Scores

The JOA instrument includes subjective symptoms, clinical examination, and activities of daily living for a total score of 29 (20). We will use the JOA instrument to observe and record the signs and symptoms scores of each participant in the two groups at each visit point. The lower the score, the more obvious the dysfunction. The JOA (21) efficacy evaluation standard will be used to evaluate overall efficacy as the primary outcome, which is the calculation of the treatment improvement rate. Treatment improvement percentage = (post-treatment score) – (pre-treatment score)/(29 –(pre-treatment score)) × 100%. An improvement rate of 100% = cured; a rate greater than 60% = effective; 25–60% = less effective; and less than 25% = invalid.

### Secondary Outcome Measures

#### Visual Analog Scale (VAS)

The VAS is a representative element of the perception of pain in the participant. It is widely used in China (22). The method uses a 10 cm long line with a continuum of 10 value points printed on one side. The two ends of the continuum are “0” and “10,” with 0 defined as no pain, and 10 defined as the worst pain imaginable.

#### Oswestry Disability Index (ODI)

The Oswestry Disability Index questionnaire (ODI) comprises 10 questions, including pain intensity, self-care ability, carrying heavy objects, walking, standing, and so on (23, 24). For each question, the participant can select from among six answer options. Each of the six selections carries a value ranging from 0 to 5, with 1 representing a low score and 5 representing a high score. The higher the score, the more severe the dysfunction.

## Sample Size and Statistical Analysis

### Sample Size

The sample size will be estimated based on a previous study that showed that an extract of thunder-fire moxibustion combined with massage eases LDH (25).

The results from a previous study showed that the mean changes in the visual analog scale (VAS) were 0.89 ± 0.37 (massage and thunder-fire moxibustion group) and 1.94 ± 0.51 (control group) (23). SPSS 21.0 software was used to calculate the sample size. The power was 90%, and the significance level was 0.05. The results from previous work showed that clinically significant differences were detectable using a sample size of 40 individuals in each group. Considering a 10% drop-out rate, we anticipate that at least 45 participants will be required in each group. Given the equality of distribution of participants between the two groups, we will recruit a total of 90 participants, with 45 in each group.

### Statistical Analysis

To eliminate artificial error, two statisticians will independently run statistical analysis *via* SPSS software (version 24.0) and the R statistical package (version 3.5.0).

Demographic and baseline data will be analyzed with standard descriptive statistics. When the baseline is unbalanced, we use the analysis using multivariate statistical methods. The JOA, VAS, and ODI scores will be analyzed using the Mann–Whitney test or Wilcoxon test. For categorical data, Fisher's exact test or the chi-square test will be implemented. Missing data will be dealt with by carrying the last observation forward. The intention-to-treat (ITT) principle will be used to analyze the main sample. If there are errors in the randomization assignment, the analysis will be performed using the assigned treatment, not the treatment that the patient received. The per-protocol population will be defined as all patients who were randomized to the thunder-fire moxibustion or the acetaminophen group and who completed the study according to the protocol.

### Safety and Adverse Events

The thunder-fire moxibustion group may encounter adverse events, including xerostomia, dizziness, smoke inhalation, scalding, and fainting. Adverse events (AEs) caused by acetaminophen mainly include acute liver failure, tinnitus, dizziness, somnolence, abdominal distension, itching, dyspepsia, and blurred vision. Should any of these adverse events occur, the researchers will diagnose whether the adverse event was caused by the treatment. Moreover, the researchers will decide whether to continue with the trial or to stop the trial and provide the corresponding compensation. The AEs caused by moxibustion will be recorded on an AEs form. These AEs will be subcategorized by severity: mild, moderate, and severe AEs (mild adverse events: adverse events that are transient and tolerable; moderate adverse events: adverse events that cause discomfort and interfere with the participant's normal life; severe adverse events: events that seriously impact the participants' physical health and might even lead to the risk of death). An AEs form will be filled in when an adverse event occurs during the treatment period, and it will include the time, duration, performance, measures to be taken, and the outcome.

### Data Management and Monitoring

A case report form (CRF) will be used for data collection. Data on demographics and assessments after each treatment will be recorded completely by the DMC for every participant. Regular monitoring tests will be conducted to ensure the authenticity of the data. The DMC was independently chaired by Genius Tianzhiyuan Group Co., Ltd., and there are no conflicts of interest. The Institute for the History and Literature of Traditional Chinese Medicine will act as an independent committee to monitor the study's progress and provide advice if necessary. The cause of patient drop-out should be clarified in the CRF for all withdrawn cases. At the end of the study, the investigator will submit the CRFs for all enrolled participants to the. Continuity of the trial will be assessed if more than 25% of the patients discontinue the interventions due to moderate or severe AEs.

The data will be double entered. Double entry of CRF data will be performed by two experienced, independent data-entry staff members within 2 weeks of data collection. The data will be stored in the final clinical trial database, which will ensure that it accurately reflects its source and meets specific quality standards.

The research and monitoring will follow the principles of good clinical practice and will be carried out by Nanling Hospital of Traditional Chinese Medicine. The clinical research assistant (CRA) will attend treatment sessions every 2 weeks to monitor and ensure the quality of the recorded data. The CRA will examine medical records, informed consent, original documents, and CRF.

## Discussion

Lumbar disc herniation (LDH) is a frequently occurring pathological condition and common spine disease in patients aged 30–50 years (26). The main manifestations are low back pain and sciatica, accompanied by the limitation of lumbar activity and changes in sensation, movement, and reflex in the affected nerve root (27). It is an adverse threat to patients' physical function or ability to work, which has a significant impact on national health care spending (28). At present, non-surgical treatment (including drug therapy and physical therapy) and surgical treatment are commonly used to treat LDH in clinics. However, surgical treatment may evoke fear of post-operative failure in patients (4). In addition, iatrogenic damage to the paraspinal muscles, ligaments, and facet joints, with reduced disc height, segmental instability, and retrolisthesis, may cause secondary pain (29).

As a type of moxibustion, thunder-fire moxibustion has stronger soft-tissue penetration than ordinary moxibustion, so it is often used to treat diseases of the digestive, (30) skeletal, (31) cardiovascular, (32) and urinary systems (33). Evidence-based studies have shown that thunder-fire moxibustion combined with acupuncture or massage has potential benefits in the treatment of LDH (34, 35). However, the current evidence cannot adequately prove the effect of thunder-fire moxibustion alone in the treatment of LDH. The purpose of this study will be to evaluate the efficacy and safety of thunder-fire moxibustion, as opposed to acetaminophen, in the treatment of patients with LDH.

During the process of moxibustion, infrared radiation will be generated (36). Far infrared waves easily penetrate the skin of the human body, and heat is transmitted and diffused, while the near-infrared waves generated can penetrate deep tissues through the capillary network. On the one hand, local heat stimulation plays a role through sensory/afferent neurons; on the other hand, it also accelerates blood circulation and promotes the rapid diffusion of local pain-causing substances (such as histamine, bradykinin, prostaglandin, etc.). However, due to the reported AEs of thunder-fire moxibustion, (37) we will take steps to prevent excessive smoke and potential scald risk. If the patient needs surgery during the trial, we will also stop the trial, and the safety or post-treatment visit can continue. We will fully respect the patient's choice, and if the patient can adhere to the treatment, we will complete the clinical trial.

Thunder-fire moxibustion at specific acupoints can eliminate local inflammation and treat low back pain. In TCM, thunder-fire moxibustion can dredge the meridians and relieve pain. In accord with the mechanisms of thunder-fire moxibustion, BL25 (Dachangshu), GV3 (Yaoyangguan), BL23 (Shenshu), and GV4 (Mingmen) were selected for inclusion in our study. The selection of these local acupoints is based on our previous clinical experience using a parallel alignment approach that may help improve the clinical effect.

Currently, the included population age range in our trial is between 25–80 years. For all participants, especially the elderly over 65 years old, we will more rigorously evaluate patient status during the trial, limit patient enrollment strictly according to the inclusion and exclusion criteria, and closely observe patient status after enrollment. After the completion of the trial, we will analyze the results according to the age as the stratification factor, and observe whether the treatment effect of thunder-fire moxibustion is different in patients of different ages.

This study has limitations. One limitation is blinding. This trial cannot be double-blind for the patients and acupuncturists, so an open-label study is used, leading to bias. However, in this study, we tried to adjust the bias by using a blind method to measure the results and statisticians' work. The trial time was short (20 days) because we focused only on the short-term analgesic effect of thunder-fire moxibustion. In addition, acetaminophen is not suitable for long-term use (38). We also refer to other clinical studies of alternative therapies for lumbar disc herniation, and the study cycle is also around 20–30 days. The previous meta-analysis (35) showed that the cycles of alternative therapy for LDH varied from 10–30 days, with an average time of around 20 days. In China, thunder-fire moxibustion is a specialized traditional Chinese medicine therapy. However, to the best of our knowledge, there are no clinical studies on the treatment of LDH with an experimental group that received simple thunder-fire moxibustion alone. Therefore, the results of randomized controlled trials on the efficacy and safety of thunder-fire moxibustion in the treatment of LDH are expected to provide high-level evidence.

## Ethics Statement

This study was approved by the Ethics Review Committee of Nanjing Hospital of Traditional Chinese Medicine (LJP No. 007). All participants will fill in informed consent forms. We will assign a special person to protect all participants' personal identifying information.

## Author Contributions

LW and HT conceived the idea for this study. JZ organized the outline and drafted the manuscript. XZ provided a few studies, ideas, and some revised opinions. XW participated in the study design and helped draft the manuscript. TX and LS contributed to the final version of the manuscript. All authors read and approved the final manuscript.

## Funding

This work was supported by Fundamental Research Funds for the Central Public Welfare Research Institutes (No. ZZ140518) and the Science and Technology Innovation Project of the China Academy of Chinese Medical Sciences (No. CI2021A00307).

## Conflict of Interest

TX was employed by Genius Tianzhiyuan Group Co., Ltd. The remaining authors declare that the research was conducted in the absence of any commercial or financial relationships that could be construed as a potential conflict of interest.

## Publisher's Note

All claims expressed in this article are solely those of the authors and do not necessarily represent those of their affiliated organizations, or those of the publisher, the editors and the reviewers. Any product that may be evaluated in this article, or claim that may be made by its manufacturer, is not guaranteed or endorsed by the publisher.

## Abbreviations

LDH: lumbar disc herniation
JOA: Japanese Orthopedic Association
VAS: visual analog scale
ODI: oswestry disability index
AEs: adverse events
NSAIDs: non-steroidal anti-inflammatory drugs
RCT: randomized controlled trial
DMC: data monitoring committee
ITT: Intention-to-treat
CRF: case report form
CRA: clinical research assistant.
